# Understanding Obesity in Individuals with Down Syndrome: Caregiver Perceptions, Awareness, and Motivation

**DOI:** 10.3390/nu18111727

**Published:** 2026-05-28

**Authors:** Thomas Cahill, Valerie Nalesso, Pat Clarke, Maria Martinez de Lagran, Andre Strydom, Li Chan, Marie-Claude Potier, Johannes Beckers, Klaus Langohr, Pietro Liò, Rafael de La Torre, Laura Forcano, Anne Hiance-Delahaye, Yann Hérault, Mara Dierssen, GO-DS21 Consortium

**Affiliations:** 1 Centre for Genomic Regulation (CRG), The Barcelona Institute of Science and Technology, 08003 Barcelona, Spain; thomas.cahill@crg.eu (T.C.); maria.martinez@crg.eu (M.M.d.L.); 2 Institut de Génétique et de Biologie Moléculaire et Cellulaire (IGBMC), UMR7104, U1258, Université de Strasbourg, CNRS, INSERM, 67400 Strasbourg, France; nalesso@igbmc.fr; 3 European Down Syndrome Association, 1210 Brussels, Belgium; 4 Institute of Psychiatry, Psychology, and Neuroscience, King’s College London, London SE5 8AF, UK; andre.strydom@kcl.ac.uk; 5 South London and Maudsley NHS Foundation Trust, London SE5 8AZ, UK; 6 The LonDowns Consortium, London SE5 8AF, UK; 7 Centre for Endocrinology, William Harvey Research Institute, Barts and the London School of Medicine and Dentistry, Queen Mary University of London, London EC1M 6BQ, UK; hhw284@qmul.ac.uk; 8 Paris Brain Institute (ICM), Pitié-Salpêtrière Hospital, 75013 Paris, France; marie-claude.potier@upmc.fr; 9 Institute of Experimental Genetics, Helmholtz Zentrum München—German Research Centre for Environmental Health, Ingolstaedter Landstr. 1, 85764 Neuherberg, Germany; johannes.beckers@helmholtz-munich.de; 10 German Centre for Diabetes Research (DZD), Ingolstaedter Landstr. 1, 85764 Neuherberg, Germany; 11 Chair of Experimental Genetics, School of Life Sciences Weihenstephan, Technische Universität München, Altekademie 8, 85354 Freising, Germany; 12 Integrative Pharmacology and Systems Neuroscience Research Group, Neuroscience Research Program, Hospital del Mar Research Institute, 08003 Barcelona, Spainrtorre2@researchmar.net (R.d.L.T.); lforcano@researchmar.net (L.F.); 13 Department of Statistics and Operations Research, Universitat Politècnica de Catalunya—BarcelonaTECH, 08034 Barcelona, Spain; 14 Department of Computer Science and Technology, University of Cambridge, Cambridge CB3 0FD, UK; pl219@cam.ac.uk; 15 CIBER de Fisiopatología de la Obesidad y Nutrición, Instituto de Salud Carlos III, 28029 Madrid, Spain; 16 Institut Jérôme Lejeune, 75015 Paris, France; anne.hiance-delahaye@institutlejeune.org; 17 PHEN—Institut Clinique de la Souris (PHEN-ICS), UAR2062, US66, Université de Strasbourg, CNRS, INSERM, 67400 Illkirch-Graffenstaden, France; 18 CELPHEDIA-Core, UAR2052, CNRS, 94800 Villejuif, France; 19 Department of Medicine and Life Sciences, Universitat Pompeu Fabra (UPF), Barcelona Biomedical Research Park (PRBB), 08003 Barcelona, Spain; 20 Biomedical Research Networking Center for Rare Diseases (CIBERER), 08005 Barcelona, Spain

**Keywords:** down syndrome, caregivers, obesity perceptions, obesity awareness, risk awareness, motivation, professional consultation, cross-sectional survey

## Abstract

Background: Individuals with Down syndrome (DS) are at an increased risk of obesity and, subsequently, its cardiometabolic and cognitive impacts. Caregivers play a critical role in managing health, yet their perceptions and behaviors have been poorly characterized. Methods: We performed a cross-sectional online survey of caregivers (*n* = 764) taking care of 48% females and 52% males with DS, conducted across European populations, predominantly in Spain and France. We assessed perceived obesity, perceived harmfulness of current weight, professional consultation, and confidence in promoting healthy behaviors. Associations were examined using chi-square tests, correlation analysis, and ordinal and logistic regression models. Results: Around one-third (32%) of caregivers perceived their family member with DS with obesity. Perceived obesity changed with age and was more frequently reported in female family members with DS. Awareness of general metabolic risk factors was high among caregivers, but half of respondents were unaware that abdominal fat affects brain health. Consultation with healthcare professionals was uncommon (57% “Never/Rarely/Sometimes”) even among those perceived with obesity. Conclusions: Caregivers demonstrate good general awareness about high energy food risks but limited knowledge of the link between obesity and brain health. Enhancing caregiver education and supporting behavioral change could promote healthier lifestyles in families with individuals with DS.

## 1. Introduction

Down syndrome (DS), or trisomy 21, is the most common chromosomal disorder worldwide, affecting around 1 in 700 to 1200 newborn [1,2,3]. It results from the presence of a full or partial extra copy of human chromosome 21 (HSA21), which leads to widespread gene-dosage imbalances and contributes to a range of developmental, physical and cognitive disturbances [4]. Individuals with DS often require lifelong caregiving support [5] and are at risk of numerous health complications, including thyroid dysfunction, gastrointestinal anomalies, and immune dysregulation, as well as an early onset of Alzheimer’s disease (AD) [6,7]. Individuals with DS are also at increased risk of obesity [8]. This is particularly concerning, not only for cardiometabolic health but also because emerging evidence links central fat distribution to poorer brain health and potentially earlier cognitive decline, a critical concern in a population already at elevated risk for AD [9,10].

Obesity is a complex, multifactorial disease, defined by a BMI ≥ 30 kg/m^2^ and characterized by excessive adiposity [11]. According to WHO, 16% of adults worldwide (890 million) are living with obesity [12]. Rates of overweight and obesity in DS are consistently higher than in the general population, with estimates ranging from 23–70% obesity in those with DS, depending on age, sex, and geography [8]. Obesity results from the interplay of behavioral and environmental factors such as poor diversified diet, excess caloric intake, and reduced physical activity [13]. Furthermore, genetic alterations associated with DS may influence insulin signaling, which have been implicated in metabolic alterations associated with type 2 diabetes, obesity, and AD [14,15]. Endocrine disturbances are also common in DS, including hypothyroidism, which slows metabolism and promotes fat accumulation [7]. Moreover, elevated circulating leptin has been reported, suggesting leptin resistance and impaired appetite regulation, further contributing to weight gain [16]. Consistent with this, a recent retrospective cohort study using U.K. electronic health records showed that people with DS have up to fourfold higher incidence of diabetes at younger ages compared to the general population [8].

Together, these factors reduce basal metabolic rate and total energy expenditure, increasing susceptibility to positive energy balance and weight gain. Increased adiposity has been detected in children with DS probably associated with poorer motor function and reduced physical activity, creating a negative feedback loop between weight gain and diminished mobility [17]. In individuals with DS, who are particularly vulnerable to obesity and its associated medical complications, caregivers and families play a central role in shaping daily routines and health-related behaviors [18]. Caregivers’ beliefs, motivations, and confidence influence whether healthy behaviors are encouraged or enacted [19]. However, there are gaps in our understanding of how caregivers perceive obesity in DS, and how they understand obesity-related health risks. Given that health-promoting attitudes are closely linked to perceptions of obesity, we aimed to evaluate caregiver beliefs and behaviors and their perceptions of obesity and its health risks to identify knowledge gaps and inform targeted interventions to improve health outcomes for individuals with DS.

## 2. Materials and Methods

### 2.1. Study Design and Participants

The objective of this study was to characterize caregiver knowledge, attitudes, and practices related to obesity in their family members with DS. To address these aims a questionnaire-based survey was developed based on the Knowledge, Attitude, Practice (KAP) framework described by Reethesh et al. [20] which was designed to help health practitioners understand obesity in patients. The instrument combined newly developed items, informed by literature review and expert input, with selected items adapted from the Obesity Awareness and Insight Scale (OASIS) (OASIS) [21], a validated Likert-scale instrument measuring obesity awareness. Survey items were grouped into KAP domains. Knowledge-related items assessed awareness of obesity-related risks such as excess sugar consumption and the knowledge of the risks of belly fat vs. overall body fat to brain health. Attitudinal responses captured caregiver perceptions of weight status and perceived harmfulness of the current weight of their family members with DS. Practice-related behaviors included consultation with doctors/dieticians for weight reduction in their family member with DS. We conducted this cross-sectional online survey that was completed by caregivers of individuals with DS (*n* ≈ 764) and was disseminated through DS associations such as EDSA, Down Spain, and Trisomie 21 France in several European countries (England, Spain, France, Germany). The survey was translated into Spanish, French, English, and German by native speakers with relevant domain expertise. Translations were reviewed collaboratively to ensure conceptual equivalence across languages, rather than literal correspondence. However, formal cross-cultural adaptation procedures (e.g., forward–backward translation or pilot testing) were not systematically conducted. The survey was anonymous and did not collect any directly identifiable personal or health data. Participants were provided with written information about the study objectives, voluntary participation, and data protection before accessing the questionnaire. Completion of the questionnaire was considered to indicate informed consent. Although the survey design was informed by established frameworks (KAP) and incorporated elements from validated instruments (OASIS), the final adapted questionnaire was not subjected to formal psychometric validation (e.g., internal consistency, construct validity). Therefore, the results should be interpreted as descriptive of caregiver perceptions rather than measurements of validated constructs.

### 2.2. Statistical Analyses

All analyses were performed in R (v4.3.2) (R Core Team (2023) [22]). Data processing and statistical analyses were conducted using the tidyverse, readxl, janitor, stringr, broom, and pscl packages. Missingness was assessed using naniar and skimr packages, and data visualization was carried out using ggplot2, scales, patchwork, and grind.

#### 2.2.1. Data Preprocessing

A cleaned dataset was generated prior to analysis, with standardized and re-coded variables. Age was presented in 2 forms (age_5, and age_4). Age_5 contained all age groups and was used for descriptive analysis. Age_4 combined the two oldest age groups (45–50 years old and >51 years into one category (>46 years)) to remove sparsity and improve model stability. Language groups were recoded to Spanish, French, English, and German, and collapsed into regional groupings (Spanish vs. Other Europe). These regional comparisons were included given the marked over-representation of Spanish-speaking participants and the potential for cultural/lifestyle differences to influence caregiver perceptions surrounding health behaviors. This grouping allowed preliminary exploration of regional exploration while also ensuring sufficient cell counts for analysis to ensure adequate sample size. Likert scale responses were retained in their original format (“Definitely”, “Probably”, “Don’t Know”, Probably not”, and “Definitely not”) for descriptive analysis and visualization. For regression analysis, responses were dichotomized into binary outcomes (Yes = “Definitely/Probably”; No = “Definitely not/Probably not”). “Don’t know” responses were excluded for regression models but retained in descriptive analysis to represent uncertainty rather than missing data.

#### 2.2.2. Missingness and Sparsity

Missingness and sparsity were evaluated prior to modelling. Missingness referred to true absent values (NA) while sparsity referred to small counts within each category. Overall, missingness for all variables used in the main analysis was minimal (<1%) and a visual inspection of missingness patterns did not show evidence of systematic missingness. Sparsity was assessed using cell counts across key variables used in regression analysis (age, sex, perceived obesity, and perceived harmfulness). Cells with <5 counts were considered sparse. Sparse cells were confined to low frequency categories (e.g., “Don’t know”) that were not included in the regression analysis. Finally, collapsing of the two oldest age groups improved cell counts and model stability. Given the low-level missingness, complete-case analysis was used for regression analysis.

#### 2.2.3. Descriptive Analyses and Data Visualization

Descriptive analyses were carried out to examine distributions of caregiver responses across demographic characteristics (sex, age group of the family member with DS, and language/regional group), caregiver perceptions, and health behaviors. These analyses were based on cross tabulations and visualized using a combination of bar charts, pie charts, and stacked bar charts (counts and percentages). To explore how different questionnaire responses related to one another, we created a series of alluvial (Sankey) diagrams. These plots allowed us to follow groups of participants across several variables at once, showing how responses clustered or shifted from one category to the next. Before generating the plots, the survey dataset was cleaned by removing unusable entries such as missing values, “Don’t know”, or “Prefer not to say” responses. Similar answer categories were then grouped together, and all variables were reordered to ensure that response levels followed a meaningful sequence. For each set of variables, we identified every unique combination of responses and counted how many participants followed each pattern. These counts formed the “flows” in the diagrams, while each response category served as a node. Flow colors were based on the first variable in the sequence to make it easier to visually track how groups moved across subsequent responses. All figures were produced in R using the ggalluvial package. This approach provided a straightforward way to visualize how caregiver perceptions, motivation, and confidence link together across different parts of the questionnaire.

#### 2.2.4. Regression Analyses

Multivariable binomial logistic regression models were used to examine associations between demographics and caregiver perceptions of obesity and harmfulness of the current weight of their family member with DS. Primary outcomes for perceived obesity and perceived harmfulness were dichotomized to “Yes” vs. “No”, defined in Section 2.2.1. Predictor variables were sex, age (age_4), and region (Spanish vs. Other Europe). Model results were reported as adjusted odds ratios (aOR), with 95% confidence intervals. We assessed model fit using pseudo-R^2^ (McFadden and Nagelkerke). “Don’t know” responses were excluded from regression models due to their ambiguous interpretation within a binary outcome framework.

## 3. Results

### 3.1. General Overview of the Survey

**Figure 1:**
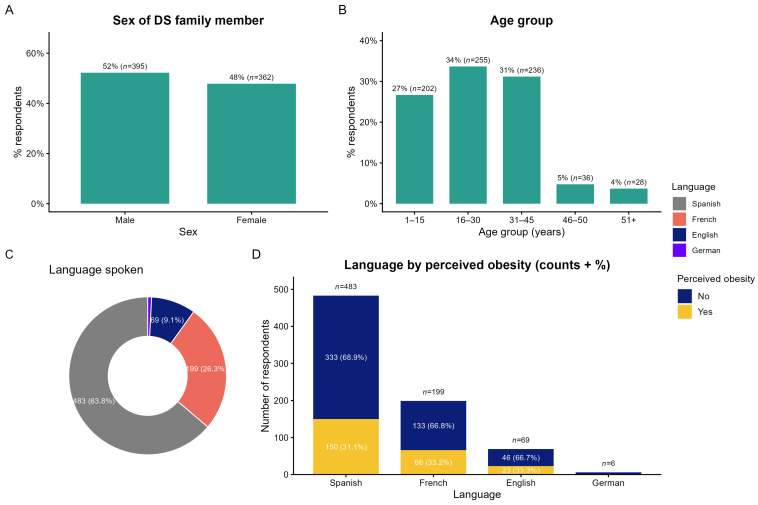
Demographics of the participants in the survey. (**A**). Proportion of sex in family members with DS. (**B**). Age of the family member with DS. (**C**). Proportion of participants speaking English, Spanish, German (n = 6 (0.8%)), and French. (**D**). Proportion of respondents per language group who perceived (Yes/Yellow) or did not perceive (No/Navy) their family member with DS as being obese. (German/No; n = 6 (100.0%)), (“Yes” = “Definitely/Probably”, and “No” = “Definitely not/Probably not”).

Responses to the survey were open-ended, and analysis showed that they were evenly balanced for sexes of the family members with DS (Figure 1A), with 52% for males and 48% for females. This distribution ensured good representativeness of both sexes in the analysis, without major gender-related bias. The most represented age groups of individuals with DS were 16–30 and 31–45 years, followed by children aged 1–15. Less than 10% of responses were for individuals with DS over 45 years old (Figure 1B). The survey, therefore, primarily includes responses referring to adolescents and young adults, a key population for lifestyle and health-related issues and an important age group for introducing interventions. Most participants were Spanish-speaking (*n* = 457), followed by French speakers (*n* = 150), with a minority of English (*n* = 69) and German speakers (*n* = 6) (Figure 1C). A summary of the demographics is displayed in Table 1.

### 3.2. Attitudes: Caregiver Perceptions of Obesity and Harmfulness

Caregiver perceptions of obesity and its perceived harmfulness represent attitudinal constructs within the KAP framework and can play a role in shaping health behaviors and intervention outcomes, particularly among vulnerable populations. This analysis examines how perceived obesity varies by age and sex, and explores the critical relationship between caregivers’ assessments of obesity and their recognition of its health risks.

#### 3.2.1. Perceived Obesity by Age and Sex

**Figure 2:**
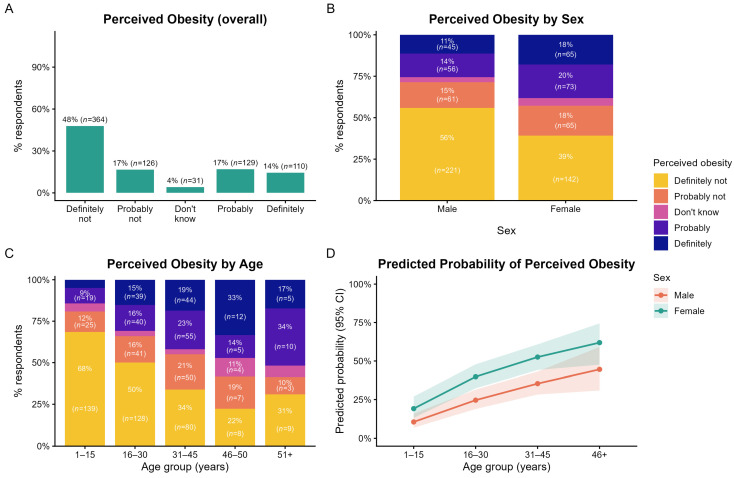
Perceived obesity according to sex and age. (**A**). Overall distribution of perceived obesity categories among respondents (“Definitely,” “Probably,” “Don’t know,” “Probably not,” and “Definitely not”), expressed as the percentage of responders selecting each category. (**B**). Distribution of perceived obesity categories stratified by sex, shown as stacked bar plots representing the proportion of male and female respondents in each category. Females are perceived more as “Definitely/Probably” obese than males (**C**). Distribution of perceived obesity across age groups illustrates age-related differences in caregiver-perception of obesity. (**D**). Predicted probabilities of perceived obesity (Yes/No) across age groups, stratified by sex. Shaded areas represent 95% CIs. Labels omitted from the figure for readability; (**B**). (Female/Don’t know; n = 17 (4.7%); Male/Don’t know; n = 12 (3.0%)); (**C**). (1–15/Definitely; n = 10 (4.9%); 1–15/Don’t know; n = 10 (4.9%); 16–30/Don’t know; n = 8 (3.1%); 31–45/Don’t know; n = 7 (3.0%); 51+/Don’t know; n = 2 (6.9%)).

Perceived obesity represents a subjective caregiver assessment of weight status rather than an objective clinical measure. Here, we report that nearly half (48%, *n* = 383) of caregivers perceived their family member with DS as “Definitely not” obese, and 17% (*n* = 126) “Probably not” obese. In contrast, only 15% (*n* = 110) considered them “Definitely” obese, and 17% (*n* = 129) “Probably” obese, while only 4% (*n* = 29) selected “Don’t know” (Figure 2A). Combined, 32% of caregivers perceived the family member with DS as “Definitely” or “Probably” obese.

Male family members with DS were more frequently perceived as “Definitely not” obese than females (56.0% vs. 39.2%) (Figure 2B). Conversely, a greater proportion of females were perceived as “Definitely” or “Probably” obese (18% and 20%, respectively), compared to males (11% and 14%).

Perceptions of obesity varied across age groups (Figure 2C). The youngest group (1–15 years) had the highest proportion of “Definitely not” obese ratings (69%) with this proportion declining across age groups, reaching 22% in the 46–50 years group. In the oldest age group (>51 years) the proportion increased to 32%, although family members older than 46 years represented only a small portion of the sample (9%). Correspondingly, the proportion of individuals perceived as “Definitely/Probably” obese increased with age through mid-adulthood.

To further examine the factors associated with perceived obesity, a multivariable logistic regression model was fitted with perceived obesity as the primary outcome with sex, age (age_4), and region as predictors (*n* = 728) (Figure 2D, Table 2). Results revealed that female family members with DS had higher odds of being perceived with obesity compared with males (aOR = 2.02, 95% CI 1.46–2.82, *p* < 0.001). Age showed a strong graded association with perceived obesity with each age group showing progressively higher odds of being perceived with obesity. Compared with individuals aged 1–15 years old, the odds of being perceived with obesity was 2.80 (95% CI 1.74–4.61, *p* < 0.001) in 16–30-year-olds, 4.69 (95% CI 2.92–7.71, *p* < 0.001) in those aged 31–45 years, and 6.88 (95% CI 3.59–13.43, *p* < 0.001) in those aged 46+ years. No association was observed for region group (Other Europe vs. Spain: aOR = 1.04, 95% CI 0.73–1.46, *p* =0.832). Model fit was modest (McFadden R^2^ = 0.078; Nagelkerke R^2^ = 0.131) (Table 2).

#### 3.2.2. Relationship Between Perceived Obesity and Harmfulness

**Figure 3:**
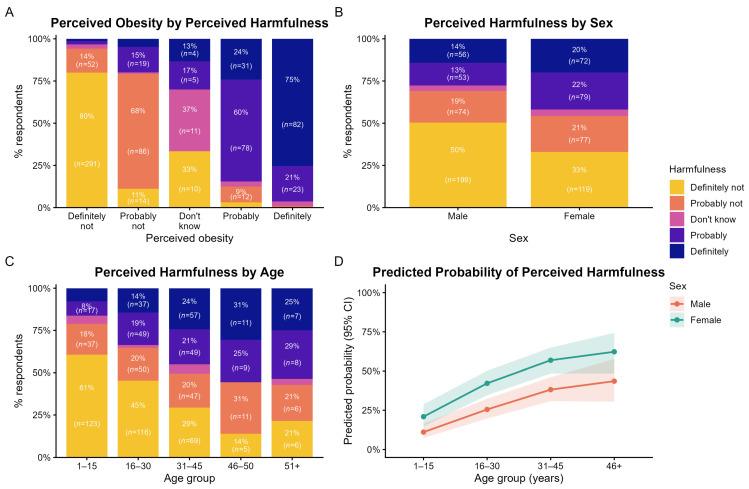
Perceived obesity and harmfulness (**A**). Shows that caregivers who perceived obesity in their family members with DS also perceived their current weight as harmful (75%). Perceived obesity categories are represented on the x-axis and perceived harmfulness of current weight is displayed as a percentage of the stacked bars in each category. (**B**). Harmfulness by sex where a higher percentage of caregivers did not think their family member’s current weight was harmful in males (50%) than in females (33%). (**C**). Harmfulness by age showing perceptions of harmfulness of current weight increases with age. (**D**). Predicted probabilities that caregivers perceived the current weight of their family member with DS as harmful (Yes/No), stratified by sex and age group. Shaded areas represent 95% confidence intervals. Labels omitted from the figure for readability; (**A**). (Definitely not/Don’t know; n = 9 (2.5%); Definitely not/Probably; n = 7 (1.9%); Definitely not/Definitely; n = 5 (1.4%)); (Probably not/Don’t know; n = 1 (0.8%); Probably not/Definitely; n = 6 (4.8%)); (Probably/Definitely not; n = 4 (3.1%); Probably/Don’t know; n = 4 (3.1%)); (Definitely/Probably not; n = 1 (0.9%); Definitely/Don’t know; n = 3 (2.8%)); (**B**). (Male/Don’t know; n = 13 (3.3%); Female/Don’t know; n = 14 (3.9%)); (**C**). (1–15/Don’t know; n = 10 (4.9%); 1–15/Definitely; n = 16 (7.9%); 16–30/Don’t know; n = 4 (1.6%); 31–45/Don’t know; n = 13 (5.5%); 51+/Don’t know; n = 1 (3.6%)).

Caregiver perceptions of obesity and perceived harmfulness showed similar patterns (Figure 3A). Among caregivers who perceived their family member with DS to be “Definitely” obese, 75% also considered their weight to be “Definitely” harmful, whereas 80% of those who perceived their family-member as “Definitely not” obese also reported that the weight was “Definitely not” harmful.

Sex-stratified analyses showed that a greater proportion of males than females were perceived as their weight being “Definitely not” harmful (50% vs. 33%; Figure 3B), consistent with earlier findings that males were more often perceived as “Definitely not” obese. Furthermore, an age-stratified analysis indicated an increase in perceived harmfulness with age, aligning with higher perceived obesity in older individuals (Figure 3C).

To further explore factors associated with perceived harmfulness we conducted a multivariable logistic regression analysis (*n* = 730) of perceived harmfulness (Yes/No) as the primary outcome with age, sex, and region as predictors. Consistent with the perceived obesity model, females were associated with higher odds of caregivers perceiving harmfulness in their current weight compared with males (aOR = 2.13, 95% CI 1.55–2.95, *p* < 0.001). Here, age also showed a strong graded association with progressively higher odds with increasing age groups relative to individuals ages 1–15 years old (16–30 years: aOR = 2.76, 95% CI 1.75–4.46, *p* < 0.001; 31–45 years: aOR = 4.99, 95% CI 3.15–8.08, *p* < 0.001; 46+ years: aOR = 6.24, 95% CI 3.34–11.88, *p* < 0.001). No significant association was observed for region (other Europe vs. Spain, aOR = 1.18, 95% 0.84–1.65, *p* = 0.349) (Table 2). Model fit was modest and comparable to that of the perceived obesity model (McFadden R^2^ = 0.084, Nagelkerke-like R^2^ = 0.142).

### 3.3. Knowledge: Awareness of Dietary and Health Risk Factors

Within the Knowledge domain of the KAP framework we assessed caregiver awareness of dietary and health risk factors, particularly the link between sugar intake, obesity, and abdominal fat.

#### 3.3.1. Awareness That Sugar Consumption Drives Obesity

**Figure 4:**
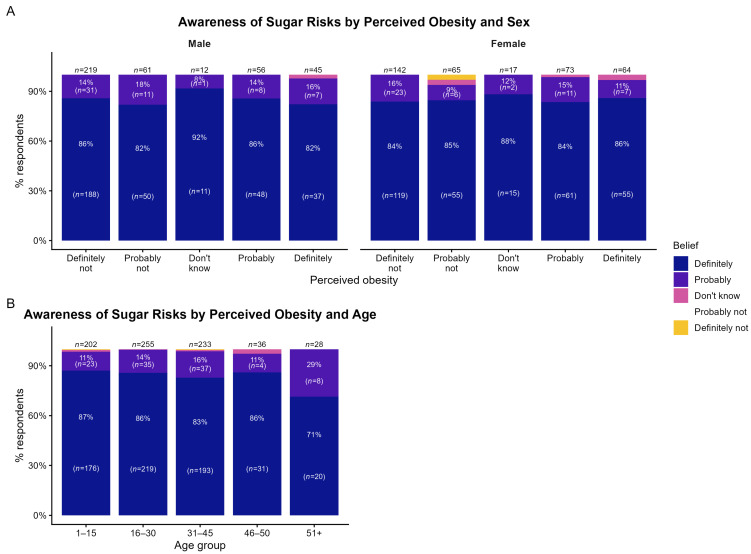
**Awareness that sugar drives obesity (by sex).** Charts of perceived obesity by belief that sugar consumption drives obesity, stratified by sex (**A**) and age (**B**). Labels omitted from the figure for readability; (**A**). (Female/Definitely/Don’t know; n = 2 (3.1%); Female/Probably/Don’t know; n = 1 (1.4%); Female/Probably not/Definitely not; n = 2 (3.1%); Female/Probably not/Don’t know; n = 2 (3.1%); Male/Definitely/Don’t know; n = 1 (2.2%)); (**B**). ((1–15/Definitely not; n = 1 (0.5%); 1–15/Don’t know; n = 2 (1.0%); 16–30/Don’t know; n = 1 (0.4%); 31–45/Definitely not; n = 1 (0.4%); 31–45/Don’t know; n = 2 (0.9%); 46–50/Don’t know; n = 1 (2.8%)).

Knowledge-related responses assessing awareness of the role of excess sugar consumption in obesity indicated high awareness across all subgroups. Figure 4A,B show that most caregivers selected “Definitely” when asked whether excess sugar contributes to obesity, regardless of perceptions of obesity, sex, or age group. Specifically, between 82% and 92% of respondents selected “Definitely” across all perceived obesity categories in both males and females, with smaller proportions selecting “Probably” (8–18%) and only a small minority selecting “Definitely not” in some categories (Figure 4A).

When examined by age group, Figure 4B shows a high proportion of respondents responding that excess sugar “Definitely” drives obesity, ranging from 87% among the caregivers of young people with DS (1–15 years) to 71% in those over 51. A decline in those selecting “Definitely” was observed among the caregivers (>51 years) of oldest individuals with DS, with 71% selecting “Definitely” and 29% selecting “Probably”.

#### 3.3.2. Abdominal Fat and Risk to Brain Health

**Figure 5:**
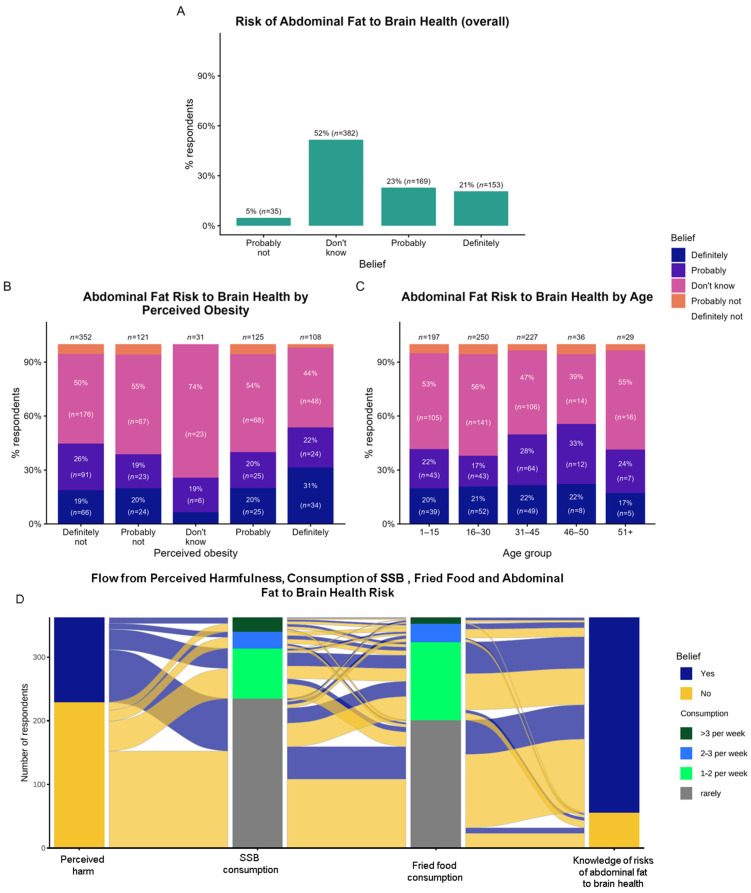
**Abdominal-fat/brain-risk belief by perceived obesity.** (**A**). Beliefs about abdominal-fat-related brain risk stratified by age group, showing limited awareness across ages, with fewer than one in four caregivers selecting “Definitely” or “Probably” agreeing that abdominal fat poses a risk to health. (**B**). Shows the distribution of caregiver beliefs that >50% answered “Don’t know” when asked whether abdominal fat poses a risk for brain health. (**C**). Beliefs about abdominal-fat-related brain risk stratified by perceived obesity status, indicating that uncertainty was common across all perceived obesity categories with most respondents answering that they “Don’t know” that abdominal fat poses a risk to brain health. (**D**). Sankey diagram illustrating the flow of caregiver responses from perceived harmfulness of current weight (Source: Yes = “Definitely/Probably” and No = “Definitely not/Probably not”) through dietary patterns of SSB consumption and fried food intake to awareness of risks of abdominal fat to brain health (destination nodes). Link width is proportional to the number of respondents following each pathway. Caregivers who perceived weight as harmful were more frequently represented in flows corresponding to lower consumption of SSBs and fried foods and greater recognition of abdominal fat risks to brain health. These Sankey diagrams are descriptive visualizations intended to illustrate co-occurrence patterns and do not imply causal or directional relationships. Labels omitted from the figure for readability; (**B**). (Definitely/Definitely not; n = 2 (1.8%); Definitely/Probably not; n = 2 (1.8%); Definitely not/Definitely not; n = 10 (2.8%); Definitely not/Probably not; n = 19 (5.2%); Don’t know/Definitely; n = 2 (6.5%); Probably/Definitely not; n = 4 (3.1%); Probably/Probably not; n = 7 (5.4%); Probably not/Definitely not; n = 5 (4.0%); Probably not/Probably not; n = 7 (5.6%)). (**C**). (1–15/Definitely not; n = 7 (3.4%); 1–15/Probably not; n = 10 (4.9%); 16–30/Definitely not; n = 5 (2.0%); 16–30/Probably not; n = 14 (5.5%); 31–45/Definitely not; n = 9 (3.8%); 31–45/Probably not; n = 8 (3.4%); 46–50/Probably not; n = 2 (5.6%); 51+/Probably not; n = 1 (3.4%)).

In contrast to sugar-related responses, our results regarding the relationship between abdominal fat and brain health indicated substantial caregiver uncertainty. Half of respondents (50%, *n* = 380) reported that they “Don’t know” whether having more belly fat is riskier for brain health than overall body fat (Figure 5A). Only 20% (*n* = 153) said they “Definitely” believed there is a link, while 22% (n = 168) answered “Probably”. Fewer respondents selected “Probably not” (5%) and “Definitely not” (3%).

When stratified by perceived obesity categories, “Don’t know” remained the most frequent response ranging from 44–76% (Figure 5C). When comparing those that viewed their family members with obesity (“Definitely/Probably” obese), 53% of respondents selected “Definitely/Probably”. This was higher than the 43% of respondents selecting belly fat “Definitely/Probably” poses a risk to brain health in those that were not viewed with obesity (“Definitely not/Probably not” obese). Those perceived as “Probably/Probably not” obese showed similar levels of certainty of the risks of brain health with 39% selecting “Definitely/Probably” affects brain health in each perceived obesity category.

Patterns were broadly consistent across age groups. Those selecting “Definitely/Probably” that abdominal fat is more harmful for brain health than overall body fat were lowest in the 16–30-year-olds (37%) followed by the over 51 group (39%), with higher proportions in mid/late adulthood (46–50-year-olds: 55%) (Figure 5C). “Don’t know” was the most frequent response across all subgroups.

Figure 5D shows co-occurrence of caregiver responses across perceived harmfulness, dietary behaviors (consumption of SSBs and fried foods), and beliefs about abdominal fat versus overall body fat risks to brain health. The flow of responses suggests that caregivers who perceived weight as harmful in their family member with DS were more frequently represented in flows corresponding to lower consumption (“Rarely”) of SSBs or fried food and greater recognition of the risks of abdominal fat to brain health.

### 3.4. Practices: Motivation to Lose Weight and Professional Consultation

**Figure 6:**
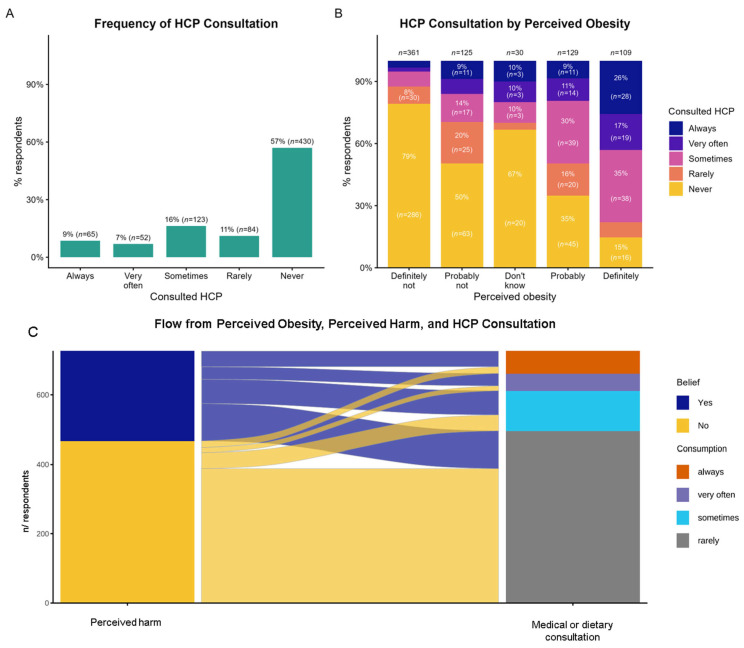
**Consultation with a health professional.** (**A**). Percentage of caregivers responding Yes/No to having consulted a doctor/dietitian regarding weight management with most having never consulted a health care professional. (**B**). Shows professional consultation by perceived obesity. (**C**). Sankey diagram illustrating the flow of caregiver responses from perceived harmfulness of current weight (Source: Yes = “Definitely/Probably” and No = “Definitely not/Probably not”)) to frequency of professional consultation for weight management (Destination: Always, Very often, Sometimes, Rarely). Link width is proportional to the number of respondents following each pathway showing that even among caregivers who perceived weight as harmful, consultation remained relatively infrequent. Labels omitted from the figure for readability; (**B**). (Definitely/Rarely; n = 8 (7.3%); Definitely not/Always; n = 12 (3.3%); Definitely not/Very often; n = 7 (1.9%); Definitely not/Sometimes; n = 26 (7.2%); Don’t know/Rarely; n = 1 (3.3%); Probably not/Very often; n = 9 (7.2%)).

Practice-related responses were assessed through caregiver-reported engagement with healthcare professionals. Caregivers were asked if they had consulted a doctor/dietician for weight reduction in their family member with DS. Overall, more than half of the caregivers reported that they had “Never” (57%) consulted a health professional regarding weight management for their family member with DS. In contrast, only 8% reported “Always” consulting a health professional with 7% choosing “Very often” (Figure 6A). When responses were stratified by perception of obesity (Figure 6B), caregivers who responded “Definitely” obese, more frequently reported consultation with a health professional (“Always” = 26%, “Very often” = 18%). In contrast, those who were perceived as “Definitely not” obese more commonly reported “Never” consulting a doctor/dietician (79%). A considerable proportion of caregivers who reported “Don’t know” for perceived obesity had reported “Never” (69%) engaging with health professionals. In addition, the flow of responses visualized in Figure 6C indicates that even among caregivers who perceived their family members’ weight as harmful, consultation was relatively infrequent.

## 4. Discussion

In this cross-sectional survey of caregivers of individuals with DS, approximately one-third of caregivers perceived their family members as “Definitely” or “Probably” obese, with perceptions varying by age and sex. Importantly, this study assessed subjective caregiver perceptions rather than objective anthropometric measures and should therefore be interpreted as subjective assessments of weight status. Perceived obesity showed similar patterns with perceived harmfulness of current weight and was descriptively related to health-related behaviors, including dietary practices and consultation with healthcare professionals. Caregivers demonstrated high awareness of the role of sugar consumption in obesity, but limited awareness of the relationship between abdominal adiposity and brain health. This is consistent with findings for the general population [23], but overall may reflect a critical gap in understanding obesity-related risks to brain health, especially important in a population already at elevated risk for early cognitive decline and AD.

Previous studies using objective anthropometric measure showed that caregiver perceptions do not consistently align with measured weight status, with underestimation frequently reported [24,25]. As the present study did not collect objective anthropometric data, it is not possible to confirm whether perceptions in the current sample reflect true weight status; the pattern of caregiver underestimation established in the literature may suggest that the 32% prevalence of perceived obesity reported here may under-represent the true burden. This remains a hypothesis that future studies incorporating objective measures would be better placed to test. Approaches such as ensuring caregiver access to resources to increase awareness in recognizing obesity and annual clinical screening have been recommended in the literature to support the early identification and management of obesity in individuals with DS [26].

### 4.1. Age and Gender Differences in Perceptions of Obesity

In the general population, BMI typically increases in mid–late adulthood and declines in older age, with slightly higher prevalence in females [27,28] and with similar patterns reported in individuals with DS [8]. In our study, caregivers were more likely to perceive female family members with DS with obesity compared with males. These sex-related patterns may reflect genuine differences in obesity prevalence and differences in caregiver perception or reporting bias, but in the absence of objective measures, these cannot be differentiated. Furthermore, large population-based studies in individuals with DS have reported age and sex-related variations in BMI with earlier peaks than the general population, that declines with age, potentially due to age-related factors [8,29]. In this study, perceptions of obesity varied across age groups, peaking in the 46+ age group. These patterns may reflect underlying age-related physiological changes, or changes in perceptions of caregivers; however, these cannot be distinguished. While further work is needed in linking direct physical measurements and perceptions of obesity, findings indicate that perceptions of obesity change across the lifespan and may be influenced by demographic factors.

### 4.2. Perceived Harmfulness of Current Weight

Perceived obesity and perceived harmfulness of the current weight showed similar patterns with caregivers who perceived their family members with obesity more frequently classifying weight as harmful. This trend mirrored the rates of perceived obesity across age groups and sex differences, where greater perceived harmfulness was seen in older age groups and in females. However, a subset of individuals perceived with obesity was not perceived as having harmful weight. Consistent with this, data on professional consultation (Section 3.4) showed that caregivers who perceived weight as not harmful were less likely to report consulting. Previous research showed that recognizing obesity may influence engagement although it can lead to psychological stresses that result in further weight gain [30]. This highlights important issues in under-recognition, and stigmatization that can impact how people view the risks associated with obesity [31,32]. The present study, however, does not allow inference on directionality or causality.

### 4.3. Awareness Gaps in Brain-Health Risks

The role of excess sugar consumption in obesity has been well documented, and strong public health messaging in recent years has focused on reducing sugar intake. This messaging has been accompanied by policy change in multiple countries such as the introduction of sugar taxes on sugar-sweetened beverages in the UK for example, contributing to high public awareness of the risks of excess sugar intake [23]. Our findings are consistent with this, showing consistently high levels of awareness that excess sugar intake drives obesity. Among the oldest age group (>51 years) and those perceived most affected with obesity, 71% selected “Definitely” compared with 87% in the 1–15 age group, representing a modest decline rather than a step-change in certainty levels.

In contrast, responses regarding the relationship between abdominal fat and brain health showed high levels of uncertainty. Several studies have demonstrated that central adiposity is associated with adverse impacts to brain volume and cognitive decline [33] and individuals with DS are at increased risk of Alzheimer’s type dementia [6]. Despite public messaging around the risks of obesity to brain health [34,35], studies have found low levels of awareness (16%) [36].

Our findings are consistent with this, showing that over half of respondents did not associate central body fat with having negative impacts on brain health. Responses also showed that awareness of this risk only slightly increased if caregivers perceived obesity in their family members with DS and peaked in mid-adulthood. Descriptively, awareness was higher in caregivers to male family members with DS, although this did not align with patterns of perceived obesity, suggesting that sex differences in awareness did not align with sex-related patterns perceived obesity. Indeed, this may suggest that campaigns need to be tailored for DS populations with communications strategies adapted for those with cognitive impairments/intellectual disabilities [37].

Furthermore, Figure 5D illustrates how KAP domains co-occur, linking perceived harmfulness (Attitudes), dietary behaviors (Practices), and awareness of the risks of abdominal fat to brain health (Knowledge). It shows that caregivers who recognized current weight as harmful were more often represented in flows to lower SSB and fried food consumption, with greater awareness of the risks of abdominal fat. This suggests that perceived harmfulness, dietary practices, and brain health awareness cluster together, rather than vary independently; however, as a descriptive visualization, the Sankey diagram cannot establish direction of these relationships. Overall, within the KAP framework, knowledge-related findings demonstrated high awareness in commonly promoted risk factors but lower awareness in risks less widely communicated.

### 4.4. Limitations

While results from this study provide informative insights into the knowledge, attitudes, and practices of caregivers to family members with DS, some limitations should be considered when interpreting the findings. First, the cross-sectional nature of the study, while sampling across different age groups, provides only a snapshot of caregiver responses at a particular point of time and does not allow for causal inference or assessment of temporal relationships. In addition, cross sectional studies are commonly used for assessing prevalence of disease; however, this study did not include objective measures of weight and thus cannot be interpreted as a clinical measure of obesity but rather the prevalence of caregiver perceptions and beliefs regarding obesity. The survey was disseminated through DS associations such as EDSA, Down Spain, and Trisomie 21 France, which may preferentially target participants engaged with support networks who may or may not be exposed to more health-related information. Additionally, the survey was self-reported and therefore open to reporting bias including social desirability bias, particularly given the stigma surrounding obesity. Furthermore, older age groups in this study were under-represented with >45-year-olds accounting for less than 10% of the total participants. The decline in survey responses for individuals with DS after age 31 may reflect a transition in living arrangements which could impact caregiver involvement and survey participation. “Don’t know” responses were excluded from the regression analyses due to their ambiguous interpretations in the binary outcomes. Although “Don’t know” responses were infrequent for key outcomes, their exclusion from regression analysis may introduce a degree of selection bias, particularly if uncertainty in perceived obesity is related to unmeasured caregiver or family characteristics. Without a sensitivity analysis to assess robustness of findings and the under-representation of these groups, care should be taken when extrapolating these results to older individuals with DS. Additionally, the pseudo-R^2^ values for the logistic regression models (McFadden R^2^ = 0.078–0.084; Nagelkerke R^2^ = 0.131–0.142) indicate modest explanatory power. As psuedo-R^2^ measures do not represent explained variance in the same way as linear-R^2^, these values should be interpreted as reflecting predictive strength rather than explained variance. This suggests that the demographic predictors included account for only a portion of the variation in caregiver perceptions, and that additional unmeasured factors are likely to contribute. In addition, the study did not collect information on caregivers, limiting the ability to assess how caregiver characteristics may influence knowledge, attitudes, and practices, or identifying sources of potential bias. Additionally, English speaking and German speaking participants were under-represented, which may affect generalizability. Finally, the questionnaire included both newly developed and adapted items that were not formally psychometrically validated. While this approach is common in exploratory KAP surveys, it limits the ability to assess reliability and construct validity and may affect cross-cultural comparability. Future studies should incorporate formal validation procedures, including pilot testing, reliability assessment, and cross-cultural adaptation protocols.

## 5. Conclusions

This cross-sectional study provides a descriptive overview of caregiver perceptions of obesity, perceived harmfulness of current weight, and health related behaviors in family members with DS. Approximately one-third of caregivers perceived their family member to be obese with perceptions varying by age and sex. Female family members with DS were more often perceived with obesity than males, and perceptions of obesity increased with age. Perceptions of obesity also showed similar patterns with perceived harmfulness. Knowledge-related findings show mixed awareness when looking at specific health risk factors. Awareness of the obesity-risks of excess sugar consumption was consistently high across all subgroups, although awareness of the risks of abdominal obesity to brain health was limited. Patterns indicate that engagement with health care professions for weight management was low, even among those who perceived weight as harmful. However, those perceived with obesity without perceiving harmfulness were less likely to seek professional help. These findings highlight the variability in caregiver knowledge, attitudes, and practices related to obesity in individuals with DS and identify areas for further research, including incorporating an objective measure of obesity and longitudinal studies to better understand how perceptions change and how they relate to health outcomes over time.

## Figures and Tables

**Table 1:** Table showing a demographic summary of the family members with DS including the total count (*n*) and percentage (%) of each sex, in each age group and of each language group.

		*n*	%
Sex	Male	391	52.1
	Female	359	47.9
Age Group	1–15	200	26.7
	16–30	255	34
	31–45	233	31.1
	46–50	36	4.8
	51+	26	3.5
Language	Spanish	482	64.3
	French	193	25.7
	English	69	9.2
	German	6	0.8

**Table 2:** **Multivariable logistic regression models for perceived obesity and perceived harmfulness.**

Model	Term	Adjusted OR (95% CI)	*p*_Value
Perceived obesity	Female vs. Male	2.02 (1.46–2.82)	<0.001
	Age 16–30 vs. 1–15	2.8 (1.74–4.61)	<0.001
	Age 31–45 vs. 1–15	4.69 (2.92–7.71)	<0.001
	Age 46+ vs. 1–15	6.88 (3.59–13.43)	<0.001
	Other Europe vs. Spain	1.04 (0.73–1.46)	0.832
Perceived harmfulness	Female vs. Male	2.13 (1.55–2.95)	<0.001
	Age 16–30 vs. 1–15	2.76 (1.75–4.46)	<0.001
	Age 31–45 vs. 1–15	4.99 (3.15–8.08)	<0.001
	Age 46+ vs. 1–15	6.24 (3.34–11.88)	<0.001
	Other Europe vs. Spain	1.18 (0.84–1.65)	0.349

## Data Availability

The anonymized survey dataset supporting the findings of this study is available in the Zenodo repository: https://doi.org/10.5281/zenodo.18863735. The survey is available at: https://seatable-ics.igbmc.fr/dtable/forms/dda98c1a-cca0-44fd-b77f-5a098217acd4/.

## Abbreviations

The following abbreviations are used in this manuscript:

KAP	Knowledge, Attitude, Practice
CI	Confidence intervals
DS	Down syndrome
RD	Risk difference
RR	Relative risk
OD	Odds ratio
WHO	World Health Organization
AD	Alzheimer’s disease
